# Visible Light-Driven Photocatalysis and Antibacterial Performance of a Cu-TiO_2_ Nanocomposite

**DOI:** 10.1021/acsomega.4c07515

**Published:** 2024-11-11

**Authors:** Michele S. de Lima, Aline L. Schio, Cesar Aguzzoli, Wellington V. de Souza, Mariana Roesch-Ely, Leonardo M. Leidens, Carla D. Boeira, Fernando Alvarez, Mariana A. Elois, Gislaine Fongaro, Carlos A. Figueroa, Alexandre F. Michels

**Affiliations:** † Postgraduate Program in Materials Science and Engineering, University of Caxias do Sul, 95070560 Caxias do Sul, Rio Grande do Sul, Brazil; ‡ Biotechnology Institute, University of Caxias do Sul, 95070560 Caxias do Sul, Rio Grande do Sul, Brazil; § “Gleb Wataghin” Institute of Physics, State University of Campinas, 13083-859 São Paulo, Brazil; ∥ Department of Microbiology, Immunology and Parasitology, Federal University of Santa Catarina, 88040-900 Florianópolis, Santa Catarina, Brazil

## Abstract

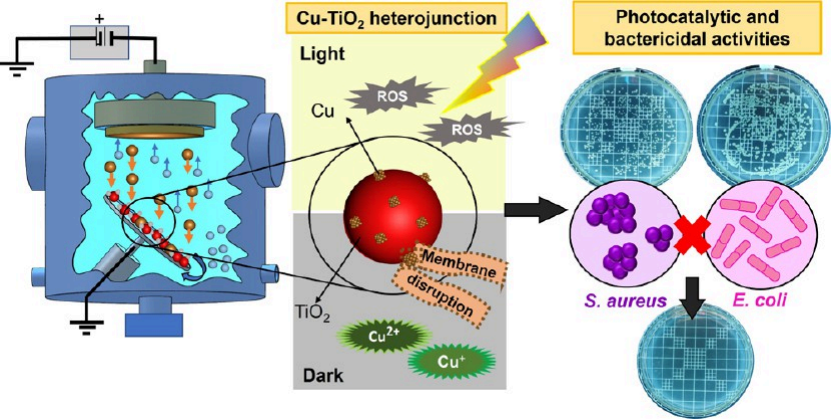

A Cu-TiO_2_ nanomaterial with unique antibacterial and photocatalytic properties is introduced in this study. Cu-TiO_2_ nanocomposites were obtained using an adapted direct current magnetron sputtering apparatus, where TiO_2_ anatase nanoparticles (NPs) were used as the substrates and copper as the sputtering target. The obtained powder was characterized by physical and chemical methods. Copper was deposited on TiO_2_ NPs for 30 and 60 min, resulting in two samples with different copper contents of 3 and 5 wt %, respectively. The photocatalysis test evaluated the degradation of rhodamine B (RhB) dye under a specific wavelength (405 nm LED) and a complete degeneration occurred in 120 min, ∼ 33% faster when compared to pristine TiO_2_. The antibacterial assays were performed for *Escherichia coli* and *Staphylococcus aureus* in dark and visible-light conditions, using a 405 nm LED and a wide-spectrum white LED, reaching an inactivation of 99.9999% for both bacteria. The magnetron sputtering is an ecofriendly way to form heterojunctions with photocatalytic and bactericidal properties in the absence of wet chemical methods or residues. This work may open new pathways for enhancing the fungicidal and virucidal activities of nanocomposites under the action of visible light.

## 1 Introduction

The COVID-19 pandemic has highlighted the urgent need to develop and enhance technologies, norms, and materials to control infections caused by different pathogens.^1^ Besides, we are exposed to various other pathogenic organisms that are present in every environment, such as hospitals, banks, supermarkets, and public transportation. These places have common-touch surfaces that can serve as potential carriers of such infectious agents.^2,3^ Microorganisms and viruses can attach to these surfaces, survive, and stay viable for infection for a long time. Therefore, limiting their continued growth on surfaces reduces the risk of infection.^4,5^ The application of coatings containing metal or metal oxide nanoparticles that act as photocatalysts would be one of the most attractive methods of disinfection.^6^ Several studies^2^ highlight that future technologies should focus on disinfection using nanoparticles (NPs) because of their inherent wide range of antiviral and antibacterial activities and efficacy at low doses. It is also pointed out that these technologies are one of the best and most cost-effective methods for combating future virus pandemics.^7,2^

A possibility of photoreactive and low-cost material (in the form of nanoparticles) for this aim is titanium dioxide (TiO_2_), which is chemically stable and presents a surface self-cleaning capacity.^8,9^ The anatase crystallographic form, which is known for its higher photoactivity than other phases,^10^ also possesses bactericidal properties that could be applied as a coating for decontaminating commonly touched surfaces.^11^ When irradiated with specific wavelengths, TiO_2_ can generate reactive oxygen species (ROS) that are responsible for oxidizing a wide range of organic compounds.^12,13^ However, due to the rapid recombination of photogenerated species and a large band gap, TiO_2_ becomes active only under ultraviolet illumination.^14,15^ Therefore, for applications in internal environments, such as door handles and other frequently touched surfaces, it is necessary to develop photoreactive materials that enhance antimicrobial activity in the visible light region.

One solution is the introduction of dopants to TiO_2_ to reduce its band gap, allowing photocatalysis to occur under visible light.^16,17^ Copper is a viable dopant for the desired application, as it absorbs visible light and exhibits excellent antibacterial activity.^18^ Photocatalysis is an established process, highly efficient, and broadly applicable for decomposing organic contaminants,^19^ compared to conventional methods.^20^ In this process, the catalyst absorbs light energy to generate an electron–hole pair, which is transferred to the surface of the photocatalyst and can initiate reactions with O_2_ and H_2_O, producing ROS that can induce damage to microorganisms and oxidize contaminants.^21,22^ The application of NPs could complement the photoresponse due to their nanoscale size, surface properties, and chemical composition.^21^

The functionalization of TiO_2_ with copper NPs is efficient in photocatalytic processes with visible light because it allows for delaying the recombination of photogenerated charge carriers in TiO_2_, due to the localized surface plasmon resonance (LSPR) effect.^23^ This effect refers to the oscillations of surface charge density of the plasmonic metals nanoparticles in resonant with an incident light wave.^21,24^ The presence of copper enables an improvement in the antimicrobial activity under visible light and sustains its activity under dark conditions due to the graduated copper ions release, as has been recently published.^25^ Also, the close contact with Cu-TiO_2_ nanomaterials and most likely formation of ROS degrade the cell membrane and promote the antimicrobial effect.^26^

In this context, fabricating Cu-TiO_2_ nanocomposites is an efficient and tested strategy for improving light absorption and enhancing photocatalytic and antibacterial activities.^27^ Amrollahi^19^ synthesized nanometal oxides modified with copper and evaluated the degradation of organic dyes under visible light illumination. Wang et al.^28^ developed a colloidal Cu-doped TiO_2_ photocatalyst with a high antibacterial rate against both *E. coli* and *S. aureus* under visible light. However, numerous works have been published using complex TiO_2_ chemical routes and the use of high-cost inputs that can make large-scale industrial applications unfeasible as well as generating a large quantity of residues and subproducts whose mechanisms are well discussed in the literature.^14^

From this perspective, physical vapor deposition (PVD) such as magnetron sputtering (MS) is a well-established industrial process, widely used to obtain multifunctional materials of high quality, avoiding the contamination of reagents, which typically occurs in other techniques.^29,30^ The achievement of a heterojunction material, through the physical deposition of Cu on a TiO_2_ substrate for photocatalytic applications has improved the photocatalytic efficiency of TiO_2_.^31^ Nevertheless, the deposition of Cu on TiO_2_ anatase NPs to generate a heterojunction with biocidal action under distinct wavelengths in the visible spectrum is still unexplored in the literature. Thus, obtaining a material via PVD with activated antipathogenic activity with visible light becomes a potential advancement, from both scientific and technological points of view.

This study aims to obtain a Cu-TiO_2_ heterojunction that has photocatalytic and bactericidal properties under illumination in the visible spectrum. For this purpose, TiO_2_ anatase NPs are functionalized with copper using a one-step process of a physical method suitable for industrial-scale application, employing an adapted magnetron sputtering system with a rotating mechanism. The effectiveness of this material, obtained by the heterojunction of copper and TiO_2_, was evaluated under dark and visible-light conditions (400–700 nm), using a 405 nm LED and a broad-spectrum white LED. The photoactivity enhancement of Cu-TiO_2_ nanocomposite is evaluated by following the degradation of rhodamine B dye and the antimicrobial activity of *E. coli* and *S. aureus* bacteria.

Finally, the results are compared with the literature, showing that the nanocomposite is effective with low lighting power. Additionally, the one-step preparation process facilitates industrial scalability.

## 2 Material and Methods

### 2.1 Sample Preparation

The samples were obtained by adapted direct current (DC) magnetron sputtering (MS) technique (Figure S1), using TiO_2_ commercial nanopowder (Sigma-Aldrich, Titanium(IV) oxide, <25 nm particle size) in anatase phase as the substrate. A solid copper target (99.9% purity) was used to deposit Cu on the TiO_2_ powder (6 cm away from the target). The deposition was conducted at room temperature, under a base pressure of 3 × 10^–6^ mbar, power of 30 W, and using argon (Air products – purity of 99.9992%) as the working gas, with a flow of 5 cm^3^·min^–1^. The deposition time was set at 30 and 60 min to obtain two samples with different concentrations, named Cu30-TiO_2_ and Cu60-TiO_2_, respectively. Lower deposition times were evaluated but were not effective in the initial tests. The equipment features a rotating system to promote the displacement of the NPs throughout the deposition process.

### 2.2 Characterization

The structural properties of the samples were evaluated by X-ray diffraction (XRD) using a diffractometer (D8 ADVANCE, Bruker) and by Raman spectroscopy (LabRAM HR Evolution, Horiba Scientific). Diffraction patterns were obtained with 2θ between 10° and 80° with Cu Kα radiation of λ = 1.5406 Å. The diffractograms were analyzed with the QualX2 software, utilizing the freely available databases.^32^ Raman spectra were acquired from 100 to 800 cm^–1^ with a laser line at 633 nm. The sample particle size distribution was mensurated by dynamic light scattering (DLS; Nanoparticle Size Analyzer NANOTRAC). The morphology of samples was analyzed by transmission electron microscopy (TEM; JEM-1011, 100 kV) and by scanning electron microscopy (FEG-SEM; MIRA3, Tescan) with a coupled energy-dispersive X-ray spectroscopy setup (EDS; SDD X-max 50, Oxford Instruments). Before imaging, all powder samples were sputter-coated with gold for a few seconds to ensure the conductivity of the surface. The copper content in the samples was quantified by ICP-OES (inductively coupled plasma optical emission spectrometry) using 3050B:1996 and 200.7:2001 methods of the Environmental Protection Agency (EPA). The optical properties of TiO_2_ anatase nanoparticles, in pristine form and after copper deposition, were recorded using a UV–vis spectrophotometer (2600i, Shimadzu) with an integrating sphere attachment. Diffuse reflectance spectra (DRS) were obtained in the range of 300–700 nm, using BaSO_4_ as a reference. The reflectance curves were further analyzed using the Tauc method and Kubelka–Munk function to estimate the band gap.^33,34^ The chemical surface was analyzed by X-ray photoelectron spectroscopy (XPS; Thermo Alpha 110 Hemispherical Analyzer with a 10 kV Al Kα radiation source), and the deconvolution procedure was performed using the CasaXPS software.

### 2.3 Irradiation Source

A homemade chamber (Figure S2) was constructed to perform photocatalytic and bacterial assays using a 405 nm LED (M405LP1; irradiance of 24.6 μW·mm^–2^) and a neutral white LED (MNWHL4; 7.7 μW·mm^–2^). All optical elements were purchased from Thorlabs Ltd. To guarantee homogeneity in the distribution of light, a focusing lens was used and the light intensity was measured with the aid of an LED light meter (Extech Instruments). For the rhodamine B test and microbiological assay for *E. coli* using a 405 nm LED, was applied a light intensity of approximately 300 and 80 lx for *S. aureus*. For the neutral white LED, a light intensity of approximately 1500 lx.

### 2.4 Photocatalysis

To evaluate the photocatalytic activity of the samples Cu30-TiO_2_ and Cu60-TiO_2_ under visible illumination the photodegradation of rhodamine B dye (RhB) (Sigma-Aldrich) was chosen as a test reaction, as reported in the bibliography.^35,36^ In 20 mL of an RhB solution (10 mg·L^–1^), 20 mg of the nanocomposite was added, and the solution was kept under agitation throughout the test inside the homemade chamber. To achieve RhB adsorption/desorption equilibrium, the solution with catalyst was maintained in the dark for 30 min and, after that, was irradiated with a 405 nm LED for 3 h. An aliquot was collected every 30 min, and centrifuged to separate the powder, and the absorbance of the supernatant was measured on a UV–vis spectrophotometer. The initial (*C*_*i*_) and final (*C*_f_) concentrations were calculated based on the calibration curve, derived from the absorbance read by the equipment.

### 2.5 Microbiological Experiments

Antibacterial tests were performed in triplicate for *E. coli* (ATCC 25922) and *S. aureus* (ATCC 25923) following the regulatory standards, with some modifications based on the bibliography.^37,38^ The minimum inhibitory concentration (MIC) and the minimum bactericidal concentration (MBC) were the concentrations of the agents needed to inhibit the visual growth or kill most of the viable organisms, respectively.^38^ MIC and MBC were defined for the samples Cu30-TiO_2_ and Cu60-TiO_2_ under dark and visible light conditions. The bacterial inoculum was adjusted to a concentration of 5 × 10^–4^ colony forming units per milliliter (CFU·mL^–1^), corresponding to 0.5 on the McFarland standard scale, and was added to different concentrations of each sample, dispersed in a 24-well plate containing tryptic soy broth (TSB) liquid medium. The plates were incubated for 24 h at 37 °C in a shaker incubator under three conditions: in the absence of light, with irradiation using a 405 nm LED and a broad-spectrum white LED. For the irradiated plates, the illumination chamber was placed inside the incubator. After 24 h, aliquots were collected from each well to determine the MIC and MBC. The MIC value was defined as the lowest concentration of the treatment at which the 0.01% w/v resazurin solution (7-hydroxy-3H-phenoxazin-3-one-10-oxide) remains blue, showing no viable cells.^39^ For the MBC, the plating was performed on a solid Mueller Hinton agar (MHA) medium, and colony counting was conducted after further incubation for 24 h at 37 °C. The inactivation efficiency was defined as log(*A*/*B*), where *A* and *B* are the number of CFU of the bacteria without exposure to treatment (positive control) and with exposure to treatment, respectively.^40^ Then, the MBC value was defined as the treatment concentration at which there was a reduction of 6 logs (99.9999%) in bacterial growth. For both the MIC and MBC, the results are presented in the form of photographic records.

## 3 Results and Discussion

### 3.1 Sample Characterization

**Figure 1:**
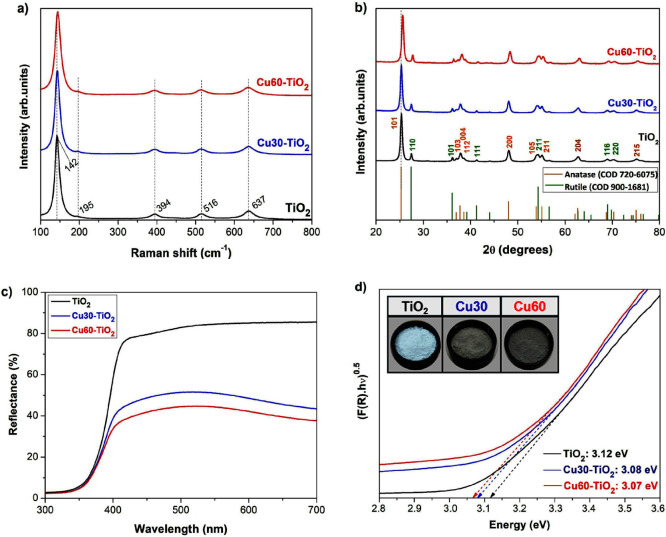
Raman shift (a), XRD patterns (b), reflectance (c) and band gap energy (d) of pristine TiO_2_ anatase, Cu30-TiO_2_ and Cu60-TiO_2_ samples (inset figure: photographs of the samples).

The Raman spectra of pristine TiO_2_ anatase nanoparticles, Cu30-TiO_2_ and Cu60-TiO_2_ are presented in Figure 1a. The peaks at approximately 142 (E_g_), 195 (E_g_), 394 (B_1g_), 516 (B_1g_), and 637 (E_g_) cm^–1^ are characteristic bands of anatase.^23^ No peaks of copper species were detected, indicating that copper could be deposited onto TiO_2_ powder, but its content is probably under the detection limit of the technique. For the sample with the highest copper concentration, a slight shift in the main peak is observed, which can be attributed to oxygen defects in the TiO_2_ network or Cu–O bonds.^23,28^ Also, in the principal E_g_ vibrational mode (at 142 cm^–1^), ascribed to symmetric O–Ti–O stretching, the small shift could indicate an expansion in the unit cell of the anatase phase.^12^ Other studies have reported that Raman peaks can undergo shifts due to surface tension acting on the structure of nanoparticles, as the surface atoms of NPs are bound by weak forces.^41^

The XRD results (Figure 1b) show the formation of anatase (COD 720-6075) and rutile (COD 900-1681) phase peaks in pristine TiO_2_ and for samples with copper deposition (Cu30-TiO_2_ and Cu60-TiO_2_). The strong XRD peaks demonstrate the high crystallinity of the anatase structure.^42^ Rietveld refinement was performed using Powdercell software,^43^ indicating the majority presence of the anatase phase (higher than 90%) (Figure S3). Similarly to the Raman analysis, characteristic peaks of copper and its species were not observed by XRD, possibly attributed to the low concentration of copper deposited by magnetron sputtering or due to the high dispersion of Cu NPs on TiO_2_.^42,44^ However, a shift of the most intense peak of the anatase phase (101) to greater angles is observed in the Cu60-TiO_2_ sample. This shift may indicate the possibility of change in the TiO_2_ lattice structure, as also observed by the reduction in the band gap that will be further discussed.^5,45^

Optical properties were evaluated for bare TiO_2_, Cu30-TiO_2_, and Cu60-TiO_2_ samples. Diffuse reflectance spectroscopy showed the increase in absorption (i.e., low reflectance) in the visible region for samples with copper (Figure 1c), thus indicating the effectiveness of the combination of copper and TiO_2_.^46^ The sharp reflectance around 400 nm was attributed to the typical band structure of TiO_2_.^31^ Liu et al.^47^ prepared TiO_2_ samples with Cu (II) nanoclusters and observed an increase in absorption in the 420–550 nm region, attributing this occurrence to an interfacial charge transfer (IFCT) process between electrons from valence band (VB) of TiO_2_ to the surface-bound Cu (II). For Kim et al.,^48^ the integration of Cu into TiO_2_ enables the IFCT process under illumination and allows accelerated photocatalytic redox reactions. According to Qiu et al.,^42^ the absorption in the range 600–700 nm could be attributed to the d-d transition of Cu (II) species and the absorption band in the range 550–600 nm is due to the interband absorption of Cu_2_O. The electromagnetic radiation in the visible range incident on the material induces collective oscillation of the free electrons. Moretti et al.,^29^ related that the presence of metallic copper can be observed by the increased absorption in the 500–650 range, which is due to a surface plasmon resonance effect.^29^

The band gap was evaluated considering TiO_2_ as a semiconductor with an indirect transition.^49^ For pure anatase TiO_2_, a band gap energy of 3.12 eV (397.4 nm) was obtained, which corresponds to values found in the literature.^50,48^ The samples Cu30-TiO_2_ e Cu60-TiO_2_ show band gaps of 3.08 eV (402.5 nm) and 3.07 eV (403.8 nm), respectively. The color of the powder samples changed from white to gray with Cu deposition (Figure 1d inset). The small reduction in the band gap was also observed by other authors for similar sample preparation conditions.^31^

Considering that the band gap values obtained for the samples containing copper correspond to 402.5 and 408.3 nm, an LED with a specific wavelength of 405 nm was used to evaluate the photocatalytic and bactericidal activities.

**Figure 2:**
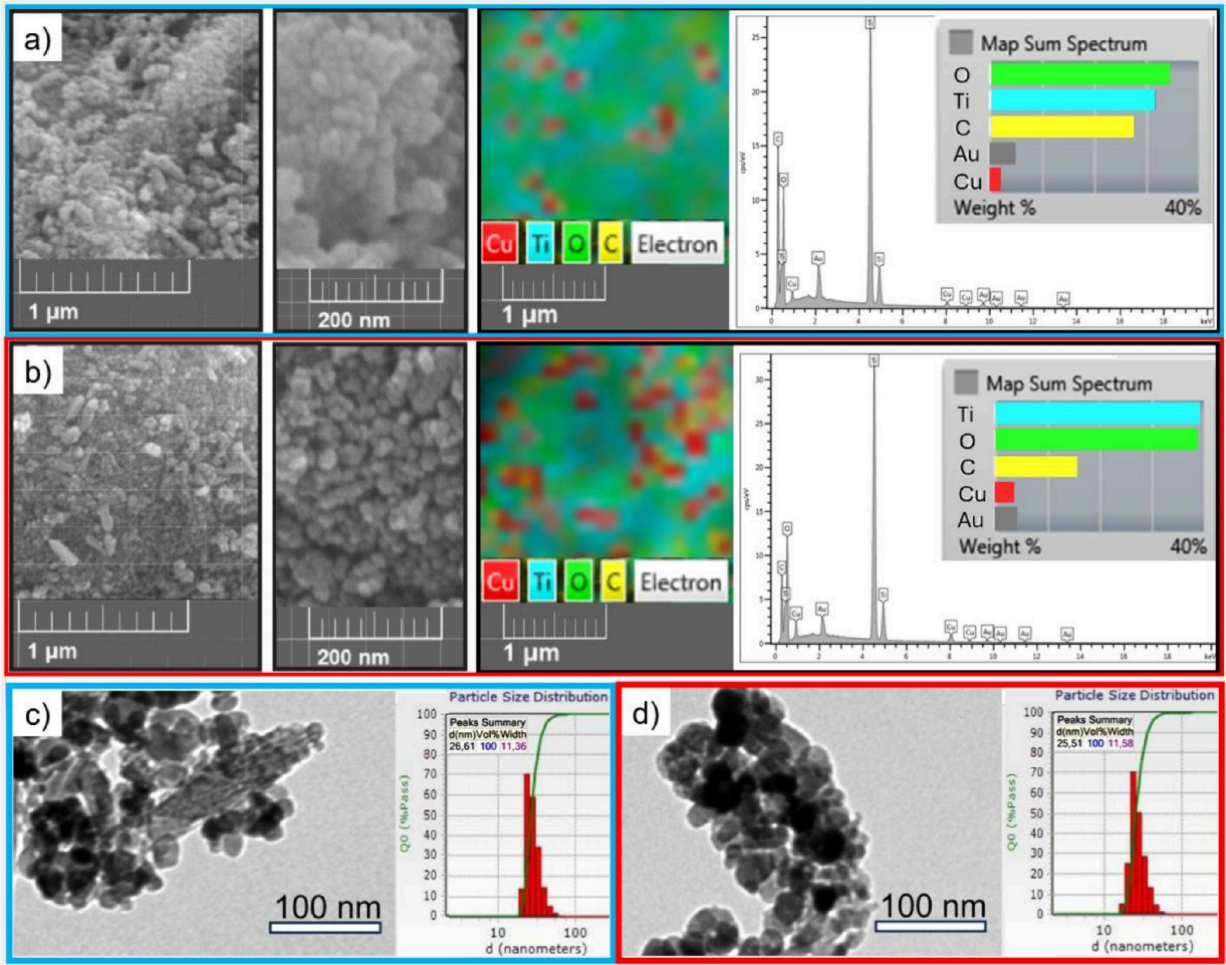
FEG-SEM images and elemental mapping by EDS for Cu30-TiO_2_ (a) and Cu60-TiO_2_ (b). TEM images and particle size distribution by DLS for Cu30-TiO_2_ (c), Cu60-TiO_2_ (d).

The FEG-SEM images with elemental mapping by EDS were recorded for the Cu30-TiO_2_ (Figure 2a) and Cu60-TiO_2_ (Figure 2b) samples. Copper, represented by the red color, is evenly distributed in the TiO_2_ NPs, in concentrations lower than 5% in both samples estimated by EDS. However, it is clear from the EDS spectra that the sample with higher deposition of Cu presents a greater density of copper clusters, as expected. The sum spectra also reveal the presence of carbon, originating from the carbon tape to fix the material, and gold, which was deposited earlier for analysis. To confirm the EDS semiquantitative observation, the concentration of deposited copper in the samples was quantified by ICP-OES, following the dissolution of nanoparticulate powder in acid media under heating. For Cu30-TiO_2_ and Cu60-TiO_2_ samples, the deposited copper concentration obtained by ICP-OES is 2.8 and 4.9 wt %, respectively, values that correspond to those previously estimated by EDS.

The TEM images with the particle size distribution are shown in Figure 2c for Cu30-TiO_2_ and Cu60-TiO_2_ in Figure 2d. TEM images show nanoparticles with spherical and polyhedral shapes with similar geometry for both concentrations. The nanoparticles particle size, mensurated by dynamic light scattering (DLS), is also similar for both concentrations, measuring approximately 26 nm for Cu30-TiO_2_ and 25 nm for Cu60-TiO_2_. As can be seen in the DLS graph results (Figure 2c,d), the particle size distribution has no evidenced aggregation status in an aqueous environment.

**Figure 3:**
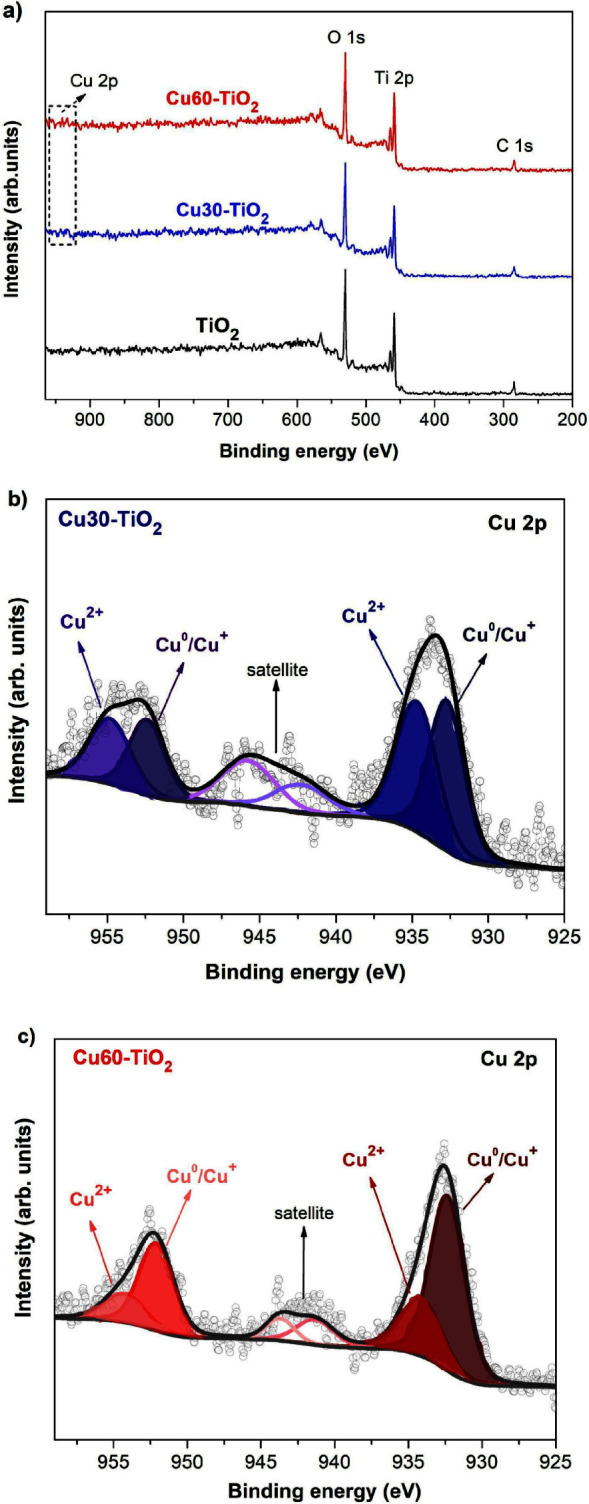
Total XPS spectra (a) for pristine TiO_2_ anatase, Cu30-TiO_2_ and Cu60-TiO_2_. XPS spectrum of Cu 2p for Cu30-TiO_2_ (b) and Cu60-TiO_2_ (c).

The chemical composition and environment of surface samples were explored by XPS. This technique is often used to examine the surface and near-surface of the material.^25^ The XPS survey spectra of bare TiO_2_, Cu30-TiO_2_, and Cu60-TiO_2_ show the presence of Ti, O, C, and Cu for the doped samples (Figure 3a). The carbon peaks are designated to the tape used as support, and the known adventitious carbon contamination is expected since the material is exposed to the ambient air.

Figure 3b, c shows the spectra of Cu 2p and its proposed deconvolutions. The peaks of the Cu^0^/Cu^+^ and Cu^2+^ species were identified in the XPS spectrum. The Cu^0^ and Cu^1+^ states have overlapping peaks, making it difficult to differentiate between them.^51^ The binding energies around 940–945 eV correspond to satellite peaks of Cu^2+^.^27^ Due to its tendency toward surface oxidation, metallic copper typically exhibits both 1+ and 2+ oxidation states.^25^

In the case of titanium, spectra of Ti 2p (Figure S4a–c) show peaks at 459.1 and 464.8 eV, which are classified as Ti^4+^–O bonds.^27^ XPS spectra of the O 1s (Figure S4d–f) showed main peaks centered at 530.3 and 531.7 eV, assigned to lattice oxide ions (Ti–O) and the Ti–OH groups on the TiO_2_ surface, respectively.^31^Table S1 presents the relative concentrations of elements in each sample. Once again, the relation of copper, comparing the 30 and 60 min deposited samples, is maintained. Additionally, in Table 1, the composition of copper and its cations obtained by XPS and ICP-OES is presented.

**Table 1:** Copper Composition Was Analyzed by XPS and ICP-OES

	XPS (at %)		ICP-OES (wt %)	
	Cu30-TiO_2_	Cu60-TiO_2_	Cu30-TiO_2_	Cu60-TiO_2_
total copper	1.5	2.5	2.8	4.9
Cu^0^/Cu^+^	50 ± 2	72 ± 2		
Cu^2+^	50 ± 2	28 ± 2		

### 3.2 Photocatalysis

**Figure 4:**
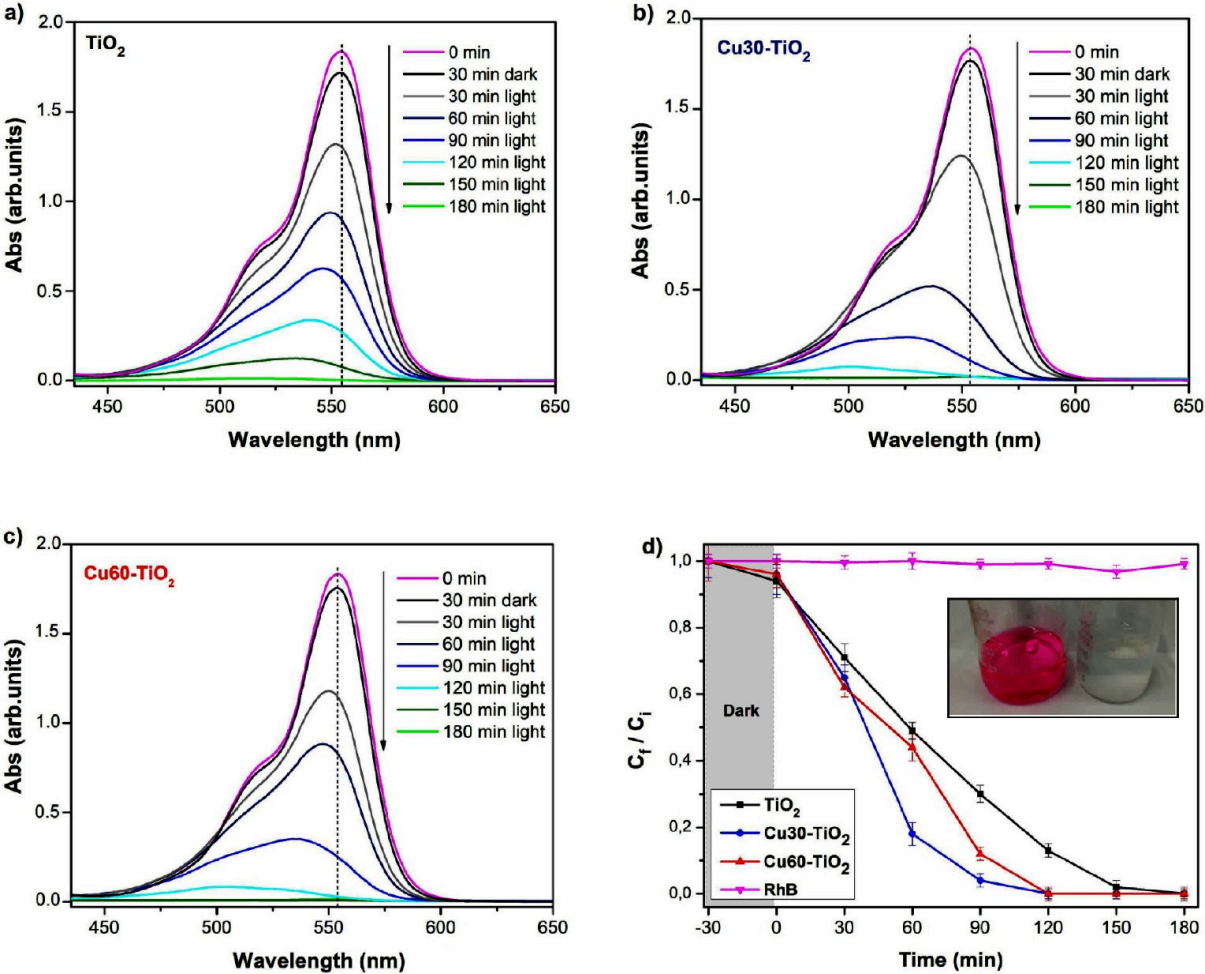
UV–vis absorption spectrum for RhB degradation with pristine TiO_2_ anatase (a), Cu30-TiO_2_ (b), and Cu60-TiO_2_ (c). *C*_f_/*C_i_* ratio versus time for pristine TiO_2_ anatase, Cu30-TiO_2_, and Cu60-TiO_2_ (d) (inset figure: photographs of the RhB solution before and after the photocatalytic process).

The reduction in the band gap energy achieved for the samples with copper deposition allowed the evaluation of photoactivity using specific wavelength illumination in the visible region of the spectrum. Rhodamine B (RhB) is widely used to study photocatalytic degradation processes due to its prevalence as a common organic dye found in industrial wastewater.^22^ Moreover, is potentially polluting, nonbiodegradable, and water-soluble, requiring its removal from aqueous solutions.^52^ The discoloration of RhB dye was monitored when in contact with photocatalysts (pristine TiO_2_, Cu30-TiO_2_, and Cu60-TiO_2_) for 3 h under visible radiation of 405 nm, as shown in Figure 4a–d. Initially, the solution exhibits a pronounced pink coloration, and photocatalytic degradation can be monitored by the decrease in absorption of the characteristic peak at 554 nm.^53^

The dye solution was exposed to light radiation without any catalyst to demonstrate that the photodegradation process occurs only in the presence of the nanomaterial (Figure 4d). When in contact with the photocatalyst, there was no significant degradation of the dye during the 30 min that the solution was kept in darkness. After 90 min under illumination, it is possible to observe that the Cu-TiO_2_ heterojunction promoted greater degradation compared to pristine TiO_2_. The degradation percentage for 90 min of reaction was 70.1% for bare TiO_2_, 88.2% for Cu60-TiO_2_, and 96.2% for Cu30-TiO_2_. After 120 min of illumination, complete degradation of the dye was achieved for samples with copper. According to the spectra (Figure 4a–c), it is observed that the intensity of the main peak (554 nm) decreases due to the cleavage of the chromophore by generated reactive oxygen species, and the peak shifts due to N-de-ethylation of RhB resulting in rhodamine-110 (absorption peak at 500 nm).^54^

Copper sputtered on TiO_2_ NPs behave as cocatalysts, improving TiO_2_ photoactivity and reducing the probability of electron–hole pair recombination.^30^ As a result, a great number of electrons reaching the surface are available to perform the reactions, increasing photocatalytic activity,^29^ and the holes generated in the valence band have strong oxidation power and are responsible for decomposing organic substances.^42^ It is widely acknowledged that photocatalysis on TiO_2_ involves the reaction of holes with surface H_2_O, to generate highly reactive hydroxyl radicals, and the reaction of electrons with O_2_ to produce highly reactive superoxide radicals.^12^ Messaadia et al.^55^ developed a Cu_2_O/TiO_2_ heterojunction using precursors and drying steps for degradation of the RhB dye under solar light irradiation (see comparative Table 2). Ding et al.^56^ developed a Cu-TiO_2_/CuO photocatalyst by in situ doping and found that this heterostructure promotes electron migration from TiO_2_ to CuO. Moreover, RhB is decomposed by the photoinduced holes on the valence band of TiO_2_, resulting in H_2_O and CO_2_.

**Table 2:** Comparison of Similar Systems for the Photocatalytic Degradation of Rhodamine B Using Cu/TiO_2_-Based Materials

method	RhB	catalyst	light source	power	maximum efficiency	reference
magnetron sputtering	10 mg·L^–1^	powder	405 nm LED	1.7 W	100% in 2h	present study
		1 mg·mL^–1^				
chemical reduction	10 μmol·L^–1^	powder	UV-A		90.1% in 2 h	(57)
		1 mg·mL^–1^				
coprecipitation and dispersion	10 mg·L^–1^	powder	solar irradiation	0.1 W·cm^–2^	100% in 2 h	(55)
		1 mg·mL^–1^				
in situ doping	10 mg·L^–1^	powder	neon lamps	120 W	96.3% in 1.5 h	(56)
		1 mg·mL^–1^				
hydrothermal	10 mg·L^–1^	powder	xenon lamp	250 W	99.4% in 1 h	(58)
		1 mg·mL^–1^				
sol–gel	10 μmol·L^–1^	nanocrystal	sodium vapor	400 W	75% in 2 h	(59)
		0.5 mg·mL^–1^				
sol–gel	10 mg·L^–1^	film	mercury lamp	9 W	100% in 1 h	(17)
		3.5 × 3.5 cm				
magnetron sputtering	5 mg·L^–1^	film	mercury lamp	300 W	92.9% in 2 h	(22)
		1 × 1 cm				

As shown in Table 2, a comparative study of similar systems for the degradation of RhB dye with Cu-TiO_2_-based materials under different light conditions is shown. As can be seen, unlike most other studies, our work used visible wavelength irradiation combined with lower power, achieving complete dye degradation in 2 h of irradiation. Additionally, we applied a one-step physical method that does not use chemical precursors or long drying and calcination times.

### 3.3 Microbiological Assays

The bactericidal effect in the absence of light and with visible illumination of the Cu-TiO_2_ heterojunction was evaluated against the bacteria *E. coli* and *S. aureus*. Both bacteria are intensely utilized to investigate the antibacterial properties of materials and are the most common pathogens causing healthcare-associated infections.^60^

**Figure 5:**
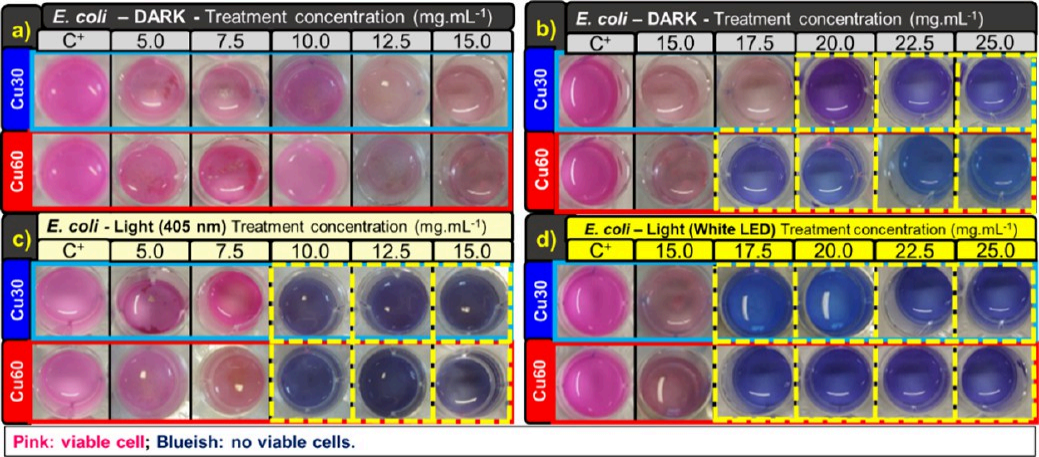
MIC results for Cu30-TiO_2_ and Cu60-TiO_2_ samples, for *E. coli* in the dark (a, b), with 405 nm irradiation (c), and with white LED irradiation (d).

**Figure 6:**
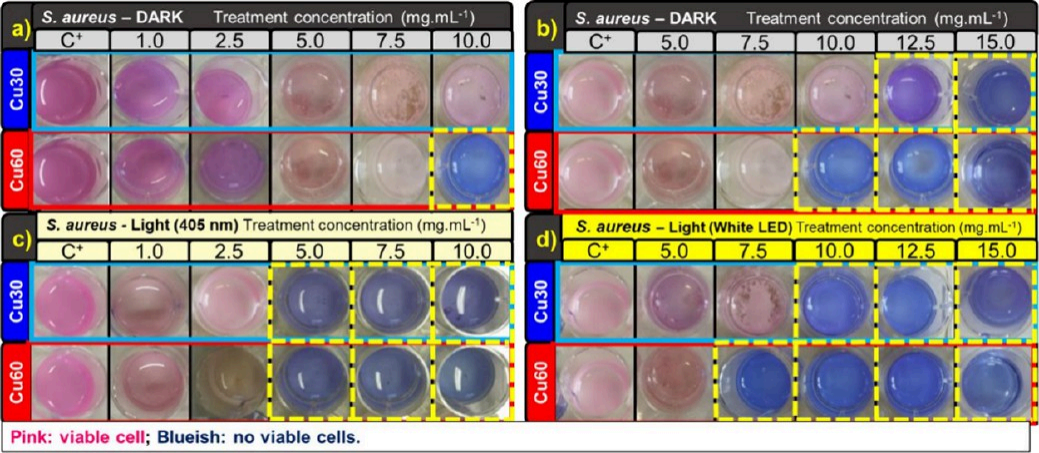
MIC results for Cu30-TiO_2_ and Cu60-TiO_2_ samples, for *S. aureus* in dark (a, b), with 405 nm irradiation (c), and with white LED irradiation (d).

**Figure 7:**
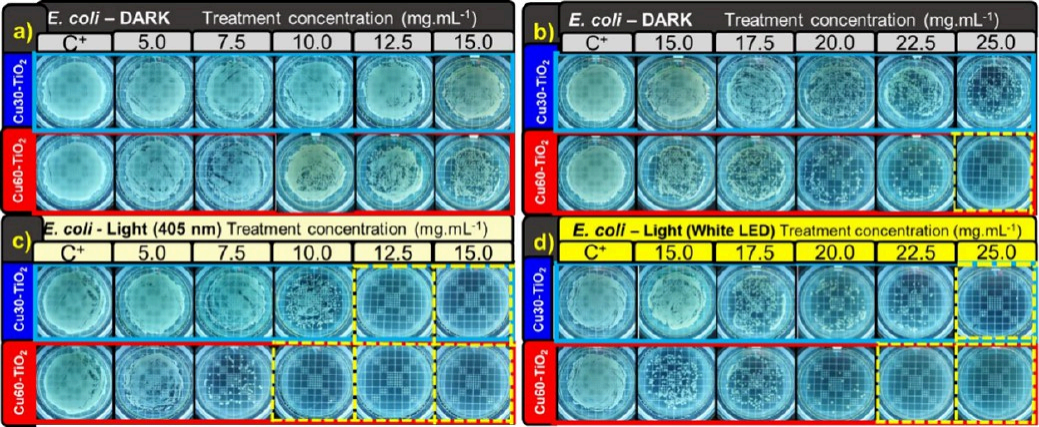
MBC results for Cu30-TiO_2_ and Cu60-TiO_2_ samples, for *E. coli* in dark (a, b), with 405 nm irradiation (c), and with white LED irradiation (d).

**Figure 8:**
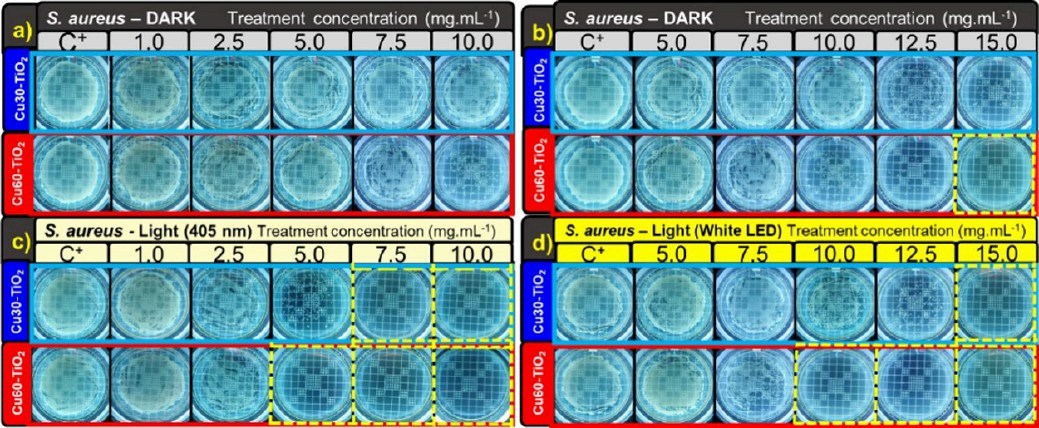
MBC results for Cu30-TiO_2_ and Cu60-TiO_2_ samples, for *S. aureus* in dark (a, b), with 405 nm irradiation (c), and with white LED irradiation (d).

Based on the band gap value found for the Cu30-TiO_2_ and Cu60-TiO_2_ samples, a specific wavelength LED (405 nm) was used to assess bactericidal activity compared to bare TiO_2_. Additionally, a broad-spectrum white LED was also applied as a visible irradiation source to demonstrate the bactericidal effect. The intensity tuned for the 405 nm LED was approximately 300 lx for *E. coli* and 80 lx for *S. aureus*. The decrease in irradiation intensity in the assay with the bacterium *S. aureus* is due to the inactivation that occurs with higher energy, observed during the tests. In other words, above 80 lx, positive control did not show bacterial growth while under illumination. In all experiments, control groups were applied, where the bacteria were not treated with any nanobactericide. For visible light illumination experiments, the control groups were also conducted under these conditions, and the respective images are shown in Figures 5, 6, 7, 8c,d.

To determine the MIC, the indicator resazurin was used to monitor the number of viable cells. When in contact with viable cells with active metabolism, the resazurin is reduced to resorufin, a fluorescent pink dye, whereas in the case of nonviable cells, the coloration becomes blueish. An adequate pink, fluorescent color is dependent on the cell type.^39^

Figure 5 presents the MIC results for Cu30-TiO_2_ and Cu60-TiO_2_ samples in five different concentrations and four conditions. In Figure 5a, it is observed that *E. coli* cells remained viable during the assay under dark conditions, for treatment concentrations up to 15.0 mg·mL^–1^, with the onset of bactericidal activity above 20.0 mg·mL^–1^ for Cu30-TiO_2_ and 17.5 mg·mL^–1^ for Cu60-TiO_2_ (Figure 5b). However, under irradiation with the 405 nm LED (Figure 5c), both samples exhibited bactericidal activity starting from 10.0 mg·mL^–1^. For irradiation with white LED (Figure 5d), both samples showed a bactericidal effect from the applied concentration of 17.5 mg·mL^–1^.

As shown in Figure 6a, it is observed that *S. aureus* cells remained viable during the assay under dark conditions, for treatment concentrations up to 7.5 mg·mL^–1^, with the onset of bactericidal activity above 12.5 mg·mL^–1^ for Cu30-TiO_2_ and 10.0 mg·mL^–1^ for Cu60-TiO_2_ (Figure 6b). Under irradiation with the 405 nm LED (Figure 6c), both samples exhibited bactericidal activity starting from 5.0 mg·mL^–1^. Under visible white LED irradiation (Figure 6d), bacteria were not viable for the Cu30-TiO_2_ sample at concentrations above 10.0 mg·mL^–1^ and above 7.5 mg·mL^–1^ for Cu60-TiO_2_. There was no significant reduction in the colony counts for the pure TiO_2_ anatase NPs under applied conditions, confirming that the material only exhibits activity under UV irradiation.

The MIC assay with the resazurin indicator complements the MBC assay, typically by showing treatment-effective concentrations that are lower or equal to those obtained through colony counting (MBC).^61^ The MBC assays for *E. coli* and *S. aureus*, in dark and visible light conditions, are presented in Figure 7. The bactericidal effect can already be observed with a decrease of ≥3 log in CFU within the specified time, indicating a 99.9% reduction in the final inoculum (resulting in 1000 CFU). A reduction of 4 log, 5 log, and 6 log corresponds to an inactivation of 99.99, 99.999, and 99.9999%, respectively.^38^ In this work, we defined the MBC value for a 6-log reduction.

Bactericidal activity in the dark against *E. coli* was observed for the Cu60-TiO_2_ sample only for concentrations above 25.0 mg·mL^–1^ (Figure 7a,b). Under the evaluated conditions, the Cu30-TiO_2_ sample had no bactericidal effect in the absence of light. However, under irradiation, the bactericidal effect occurred for lower concentrations of each sample, i.e., 12.5 mg·mL^–1^ (405 nm LED, Figure 7c) and 25.0 mg·mL^–1^ (white LED, Figure 7d) for the sample Cu30-TiO_2_ and 10.0 mg·mL^–1^ (405 nm LED, Figure 7c) and 22.5 mg·mL^–1^ (white LED, Figure 7d) for the sample Cu60-TiO_2_.

The microbiological assays with *S. aureus* resulted in a 6-log reduction only for the Cu60-TiO_2_ sample and at concentrations above 15.0 mg·mL^–1^ under dark conditions (Figure 8a,b). For the Cu30-TiO_2_ sample, it is possible to observe the onset of bactericidal activity at a concentration of 12.5 mg·mL^–1^ due to the apparent reduction in the number of colony-forming units (Figure 8b). However, under illumination, the Cu30-TiO_2_ sample reached a 6-log reduction in CFU at concentrations of 7.5 mg·mL^–1^ with 405 nm (Figure 8c) and 15.0 mg·mL^–1^ with a white LED (Figure 8d). For the Cu60-TiO_2_ sample, at 5.0 mg·mL^–1^ with 405 nm LED (Figure 8c) and 10.0 mg·mL^–1^ with broad-spectrum visible LED (Figure 8d) there is no colony growth. As an MIC assay, there was no significant reduction in the colony counts for the pure TiO_2_ anatase NPs in applied conditions, confirming that the material only exhibits activity under UV irradiation (Figures S5 and S6). The results obtained confirm the increased bactericidal activity of the Cu-TiO_2_ heterojunction under visible illumination. The MIC and MBC results are summarized in Table S2.

As shown in Table 3, a comparative study of similar systems for bactericidal activity against *E. coli* and/or *S. aureus* using Cu-TiO_2_-based materials under distinct light conditions is presented. Unlike most other studies, the present work combined visible wavelength irradiation with lower power, achieving a maximum reduction efficiency of a 6-log reduction for *E. coli* and *S. aureus*. Additionally, the method of obtaining the nanocomposite involved a physical one-step process, making it easy to scale up industrially.

**Table 3:** Comparison of Similar Systems for Bactericide Photoactivity Against Bacteria Using Cu/TiO_2_-Based Materials

method	bacteria	catalyst	light source	power	maximum efficiency	reference
magnetron sputtering	*E. coli* and *S. aureus*	powder	LED (405 nm)	1.7 W	6-log	present study
			LED (white)	0.88 W		
hydrothermal	*E. coli*	powder	UVA	10 W	>6-log	(62)
chemical deposition	*E. coli* and *S. aureus*	film	UVA	16 W	<6-log	(12)
photodeposition	*E. coli*	powder	Xe lamp	450 W	>6-log	(63)
alkaline-heat treatment	*E. coli*	film	LED (460 nm)	250 W/m^2^	<6-log	(64)
hydrothermal	*E. coli* and *S. aureus*	colloidal	fluorescent lamp	1000 lx	5.96-log	(28)
sol–gel	*E. coli* and *S. aureus*	powder	visible light		5-log	(50)

**Figure 9:**
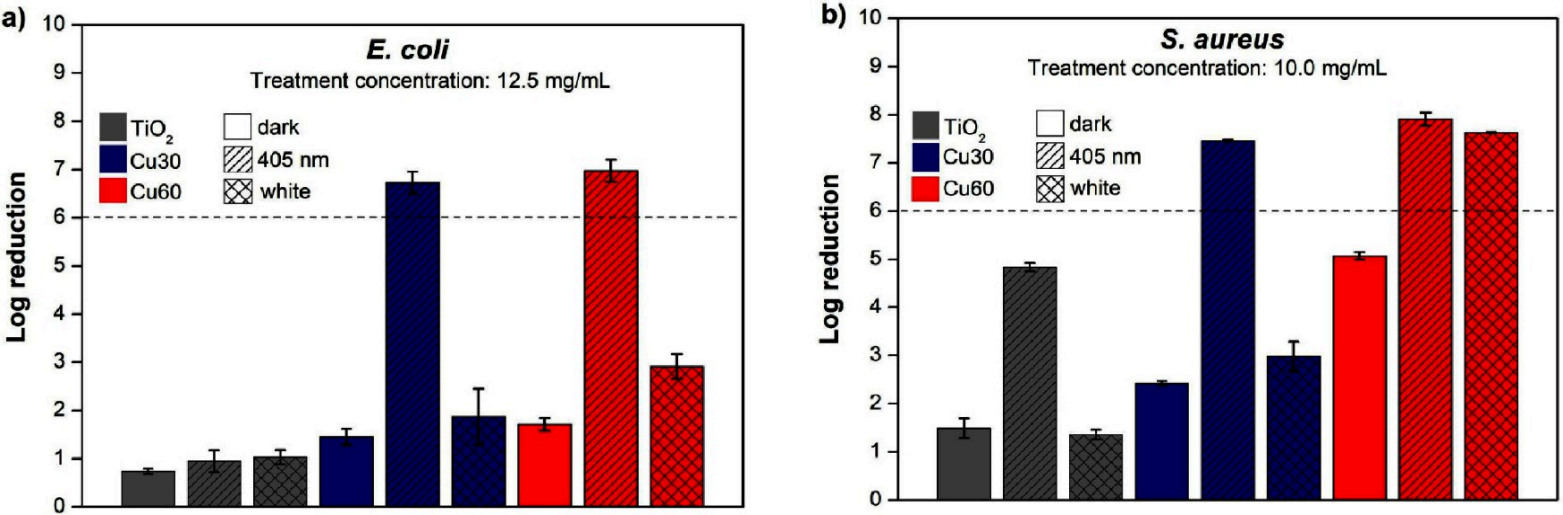
Log reduction graphs for TiO_2_, Cu30-TiO_2_ and Cu60-TiO_2_ for *E. coli* (a) and *S. aureus* (b) under dark and visible light conditions. The log reduction results were obtained in comparison with the control (without nanomaterial and under dark conditions). The dashed lines are guides for the eye.

Figure 9 illustrates the comparison of the logarithmic reduction for TiO_2_ anatase, Cu30-TiO_2,_ and Cu60-TiO_2_ samples at concentrations of 12.5 mg·mL^–1^ for tests with *E. coli* (Figure 9a) and 10.0 mg·mL^–1^ with *S. aureus* (Figure 9b) under dark and visible illumination (405 nm and broad-spectrum white LED). It can be observed that the bactericidal effect increases for the samples containing copper and when the tests occur under irradiation. Evaluations with control groups where the bacteria were not treated with any nanobacterial agent but only with different lighting conditions were performed to compare the log reduction with the control under dark conditions (Figure S7a,b). For both bacteria and distinct wavelength conditions, there was a difference of less than 1-log, maintaining the 6-log reduction for the samples under their specific conditions.

According to previous studies, a small but significant decrease in the number of colonies can occur under dark conditions for both bacteria, due to the cytotoxicity of copper species.^12^ Besides that, the solid-state Cu_*x*_O nanoclusters and Cu^1+^ species induce antimicrobial activity.^42^ The absorption or the direct contact of the particles with the bacteria can cause their inactivation under dark conditions.^65^ The precise mechanisms underlying the greater effectiveness of inhibiting the growth of Gram-positive bacteria such as *S. aureus* compared to Gram-negative bacteria like *E. coli* are not completely understood. However, it is speculated that this difference could be attributed to the increased permeability of the cell envelope of Gram-positive bacteria by cytotoxic Cu ions. Gram-positive bacteria possess a highly permeable cell wall, which may facilitate the entry of Cu ions. In contrast, although Gram-negative bacteria have a thinner cell envelope, it comprises two membrane layers serving as effective barriers to permeability.^21,12^

The higher activity of samples with copper compared with pristine TiO_2_ anatase could be attributed to states of Cu that function as trapping sites, reducing charge carrier recombination. These photogenerated charges travel to the surface of the catalyst and react with water and oxygen to produce ROS. Also, the presence of copper allows antibacterial activity by Fenton-type reactions.^12^ Both pathways, by Fenton mechanism or photocatalysis, lead to the production of hydroxyl radicals.^25^ For Qiu et al.,^42^ the irradiation with visible light can generate holes in the TiO_2_ valence band and Cu^+^ species that could contribute to the antipathogenic effect. The ROS are also a byproduct of cellular metabolism; however, in excess, they can induce damage to cells and their structures. With the increase in the concentration of the Cu-TiO_2_ catalyst, the content of ROS produced by bacteria can increase due to more active sites, leading to a decrease in the bacterial load.^44^ Other studies revealed that Cu (I) oxides adsorbed and denatured proteins to a greater extent than Cu (II) oxides upon direct contact.^27^

Several mechanisms for the decomposition of organic compounds and antimicrobial activity using Cu/TiO_2_ association materials under visible light irradiation have been suggested. These include copper doping,^56,28^ plasmonic NPs (LSPR),^23^ and another type of heterojunctions.^55^ It has been proposed that those mechanisms can occur simultaneously, as they are highly dependent on the properties of the photocatalyst.^63^ There is still no consensus in the literature regarding the exact mechanism that leads to bacterial death, as it depends on the material used for treatment, the size and shape of this material, the conditions under which the assays were conducted, the type and intensity of irradiation, and the bacterial strains.

**Figure 10:**
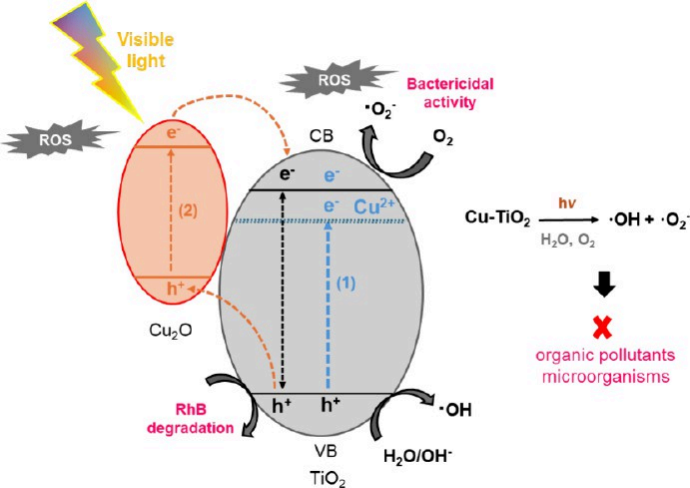
Suggested mechanism for the synergistic photocatalysis of RhB degradation and bactericidal activity in Cu30-TiO_2_ and Cu60-TiO_2_ samples under visible light illumination.

A scheme, presented in Figure 10, is proposed to explain two possible pathways for the mechanism responsible for the degradation of RhB dye and bactericidal activity under visible light, based on the results obtained. When the band gap is reduced by copper doping (1), an additional midgap electronic level is created and the absorption range in the visible spectrum is extended. Electrons from this intermediate energy level and the valence band of TiO_2_ migrate to the conduction band of TiO_2_, leading to the formation of ROS and enhanced photocatalytic activity.^56,28^ In the second pathway (2), under visible light illumination, the electrons are transferred from the CB of Cu_2_O to the CB of TiO_2_, and the photogenerated holes migrate in opposite directions. The interface between Cu_2_O and TiO_2_ creates a concentration of charge carriers that will react with oxygen and water to produce ROS.^50,66^

The results presented in this work aim to demonstrate the bactericidal effect of the Cu-TiO_2_ heterojunction prepared via magnetron sputtering. Greater photocatalytic and bactericidal activities were observed under visible light conditions compared with dark conditions and pure TiO_2_. Under illumination, the copper NPs in metallic and oxidized forms can create a heterojunction with titania, acting as trapped sites for photoinduced electrons, reducing photogenerated carrier recombination, and enhancing the photocatalytic properties of TiO_2_.^29^ We observed that a synergetic effect between copper (metallic copper and copper species) and anatase TiO_2_ NPs could explain the best photocatalytic and bactericidal activities of the samples prepared using the DC-MS technique. The bactericidal effect in dark conditions could be explained by the release of copper ions and close contact with the nanoparticles, which will disrupt the cell membrane, growing with the increase of copper content. Thus, when light irradiation is present, ROS formation occurs, which will enhance the bactericidal activity.

## 4 Conclusions

Direct current-adapted magnetron sputtering was used to deposit copper on the TiO_2_ anatase NPs. The Cu loading content increased with deposition time (30–60 min), resulting in copper concentrations of approximately 3 and 5% for Cu30-TiO_2_ and Cu60-TiO_2_ samples, respectively. FEG-SEM, TEM, and DLS revealed particles in the nanometer size range (about 26 nm), with spherical and polyhedral shapes. UV–vis spectroscopy showed a slight reduction in the band gap energy for the samples prepared by the sputtering process. Complete degradation of the rhodamine B dye was reached for Cu30-TiO_2_ and Cu60-TiO_2_ samples in 2 h with 405 nm LED irradiation, while it took 3 h for bare TiO_2_. The bactericidal activity was evaluated against *E. coli* and *S. aureus*, showing increased effectiveness with higher copper concentrations and visible light irradiation, resulting in a 6-log reduction in colony-forming units. In conclusion, the physical deposition of Cu on a TiO_2_ nanopowder support demonstrates promising photocatalytic activity in degrading rhodamine B as well as a significant bactericidal effect. This combination of characteristics suggests their potential application in environmental remediation and disinfection processes. However, depending on the nanoparticle dispersion process, cluster formation can be a source of variability in the efficiency of this nanocomposite. Future studies may evaluate the fungicidal and virucidal activities under visible light on functionalized surfaces with this TiO_2_-based nanocomposite.

## Supporting Information Available

Additional experimental details including: figures of experimental setup for magnetron sputtering equipment (Figure S1) and the homemade illumination chamber (Figure S2); Rietveld refinement for TiO_2_ NPs (Figure S3); XPS survey spectra for bare TiO_2_ NPs, Cu30-TiO_2_ and Cu60-TiO_2_ samples (Figure S4); elements composition according to XPS (Table S1); MBC assay with *E. coli* and *S. aureus* for pristine TiO_2_ NPs in dark and visible light conditions (Figures S5 and S6); MIC and MBC values for Cu30-TiO_2_ and Cu60-TiO_2_ samples under dark or visible conditions (Table S2); log reduction results under visible light conditions (Figure S7) (PDF)

## Abbreviations

ATCC: American-type culture collection
CB: conduction band
*C_i_*: initial concentration of RhB
*C*_f_: final concentration of RhB
CFU: colony-forming unit
DC-MS: direct current magnetron sputtering
DLS: dynamic light scattering
DRS: diffuse reflectance spectra
EDS: energy-dispersive spectroscopy
EPA: Environmental Protection Agency
ICP-OES: inductively coupled plasma optical emission spectrometry
IFCT: interfacial charge transfer
LED: light-emitting diode
LSPR: localized surface plasmon resonance
MBC: minimum bactericidal concentration
MHA: Mueller Hinton agar
MIC: minimum inhibitory concentration
NPs: nanoparticles
PVD: physical vapor deposition
RhB: rhodamine B
ROS: reactive oxygen species
SEM: scanning electron microscopy
TEM: transmission electron microscopy
TSB: tryptic soy broth
VB: valence band
XRD: X-ray diffraction
XPS: X-ray photoelectron spectroscopy
