# Supplementary material for: Visible Light-Driven Photocatalysis and Antibacterial Performance of a Cu-TiO2 Nanocomposite
Source: ACS Omega. 2024 Nov 11;9(47):47122–34. doi: 10.1021/acsomega.4c07515 (PMC11603235; doi:10.1021/acsomega.4c07515)
Supplement: Supplementary file 1 — ao4c07515_si_001.pdf [file ao4c07515_si_001.pdf]

Supporting information

Visible light-driven photocatalysis and antibacterial performance of a Cu-TiO_2 nanocomposite

Michele S. de Lima[1], Aline L. Schio[1], Cesar Aguzzoli [1], Wellington V. de Souza[2], Mariana Roesch-Ely[1,2], Leonardo M. Leidens[3], Carla D. Boeira[3], Fernando Alvarez[3], Mariana A. Elois[4], Gislaine Fongaro[4], Carlos A. Figueroa[1], Alexandre F. Michels[1*]

[1] Postgraduate Program in Materials Science and Engineering, University of Caxias do Sul, 95070560 Rio Grande do Sul, Brazil.
[2] Biotechnology Institute, University of Caxias do Sul, 95070560 Rio Grande do Sul, Brazil
[3] "Gleb Wataghin" Institute of Physics, State University of Campinas, 13083-859 São Paulo, Brazil
[4] Department of Microbiology, Immunology and Parasitology, Federal University of Santa Catarina, 88040-900 Santa Catarina, Brazil

Corresponding author

Alexandre F. Michels; email: alexandrefassinimichels@gmail.com

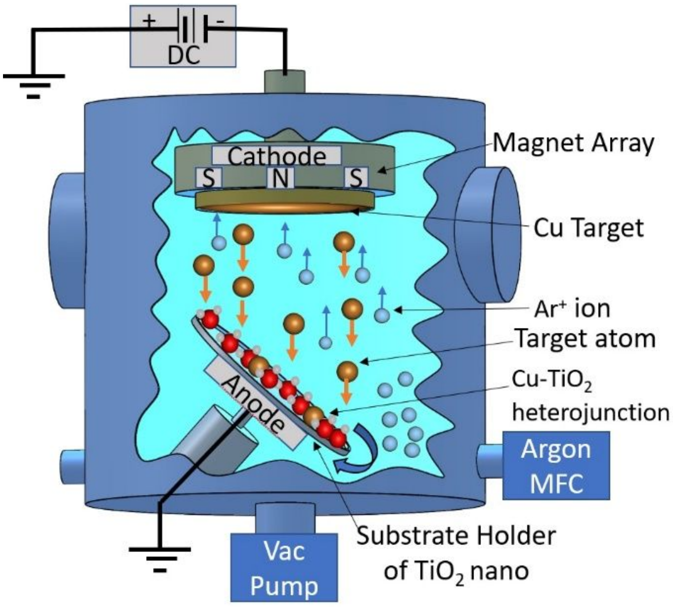

Fig. S1. Direct current adapted magnetron sputtering equipment.

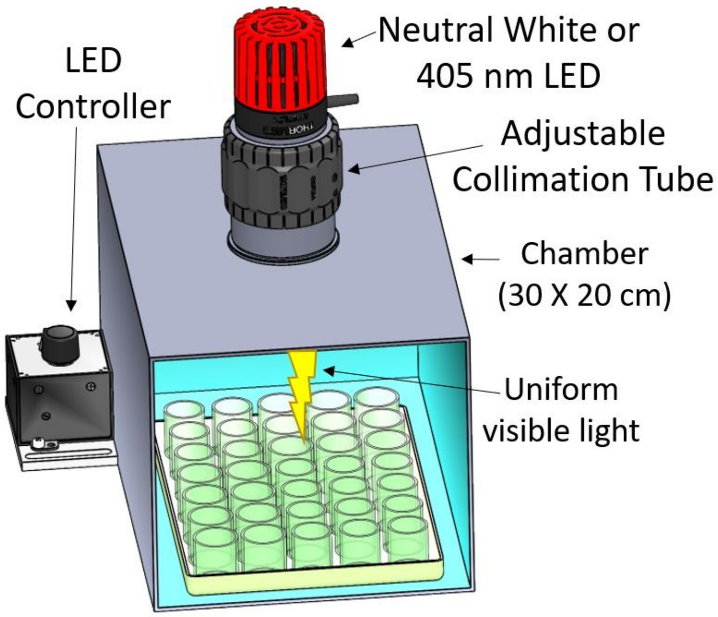

Fig. S2. Homemade illumination chamber with ThorLabs devices.

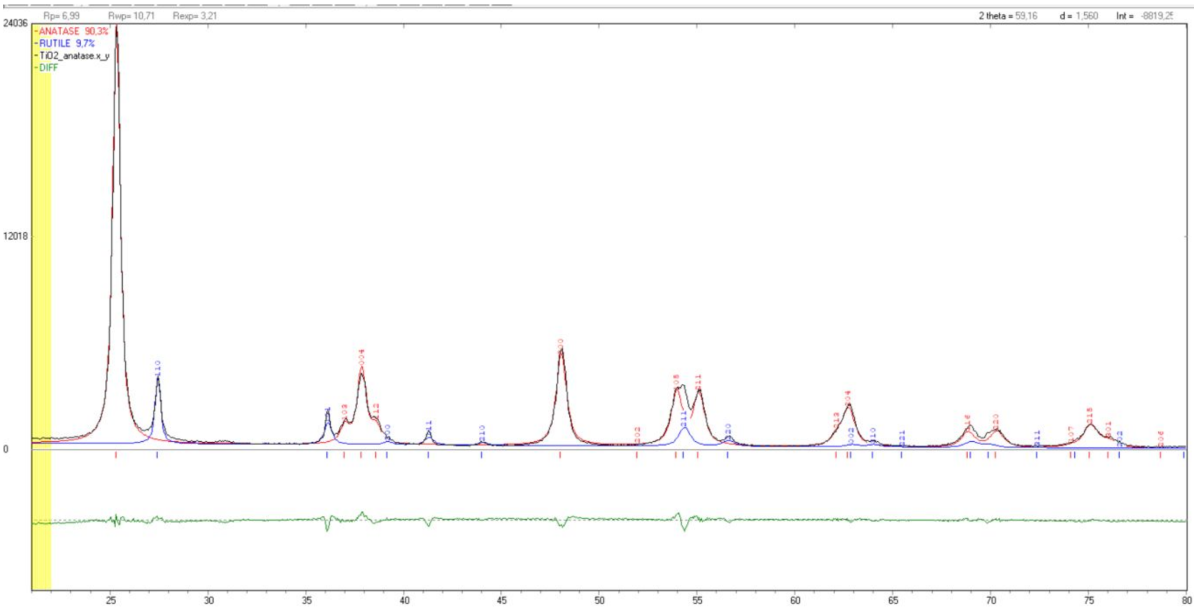

Fig. S3. Rietveld refinement for anatase TiO_2 NPs obtained with Powdercell software.

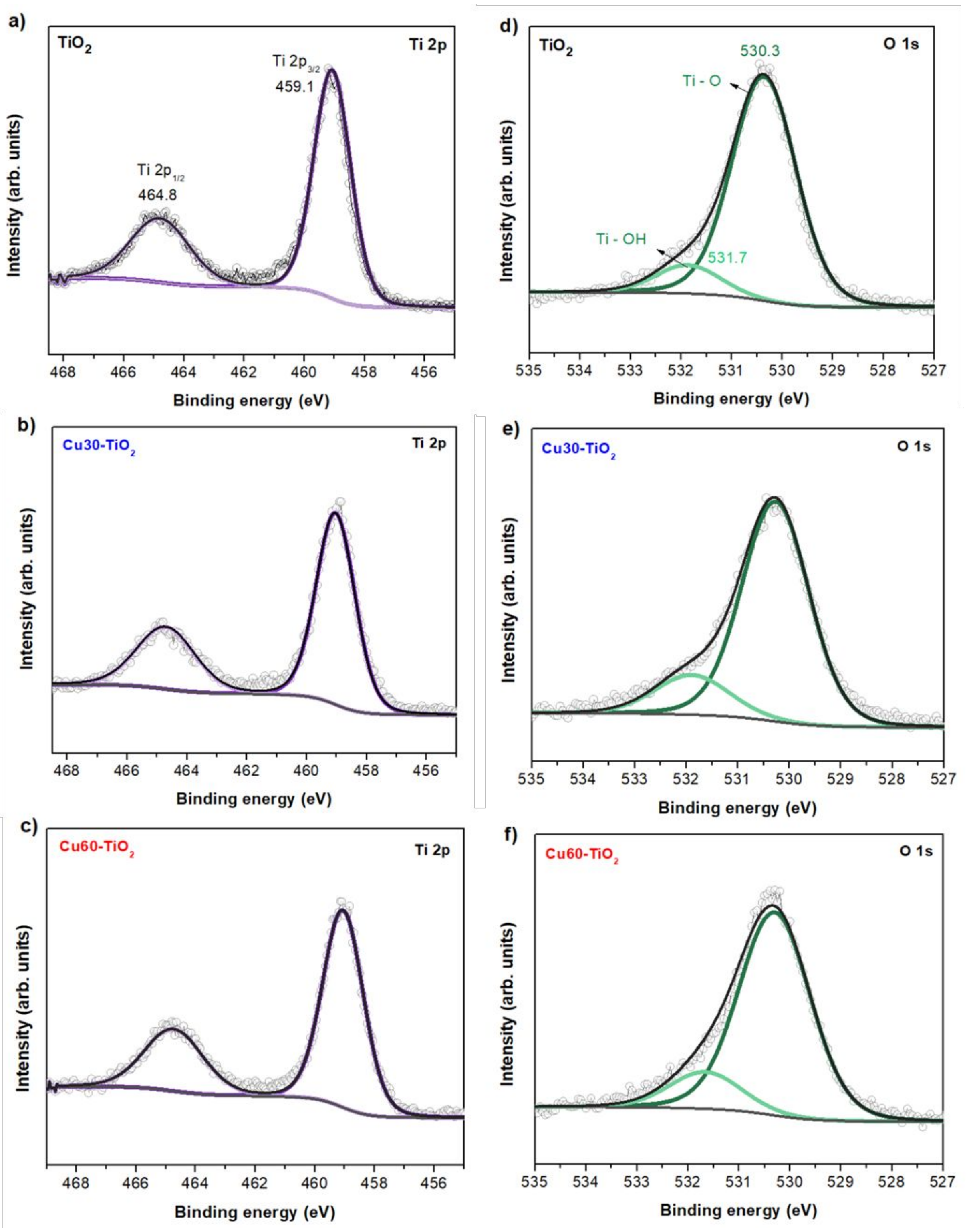

Fig. S4. XPS survey spectra of Ti 2p (a-c) and O 1s (d-f) for bare TiO_2 anatase, Cu30-TiO_2 and Cu60-TiO_2

Table S1. Elements composition (wt.%) according to XPS data for bare TiO_2, Cu30-TiO_2 and Cu60-TiO_2. The uncertainty of concentration is in the order of 10 % to all the elements.

Element	TiO_2 anatase	Cu30-TiO_2	Cu60-TiO_2
Ti 2p	22.5	21.5	20.0
Cu 2p	0.0	1.5	2.5
O 1s	55.0	54.0	54.5
C 1s	22.5	23.0	23.0

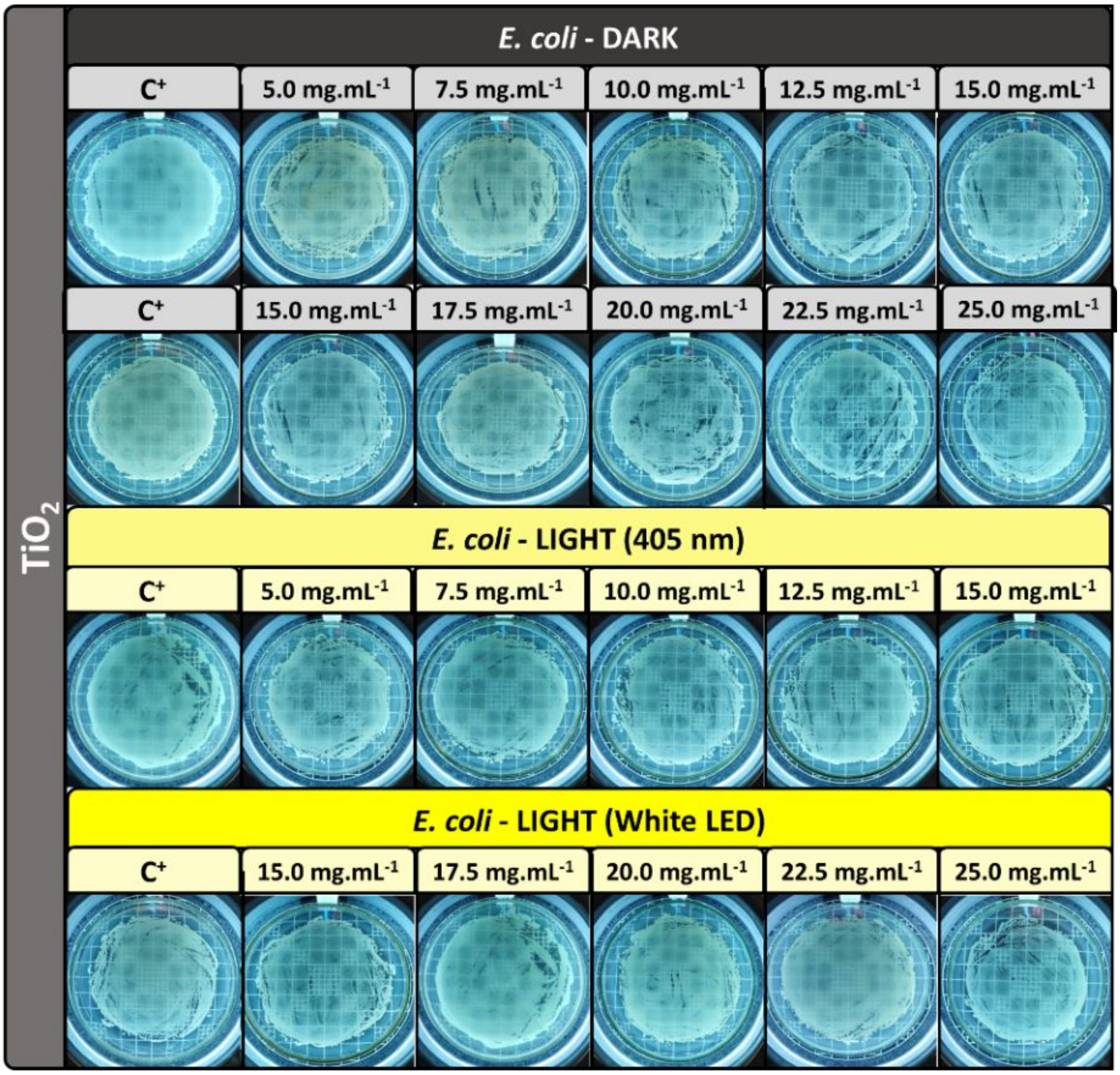

Fig. S5. MBC assay with *E. coli* for pristine TiO_2 anatase NPs in dark and visible light conditions (405 nm and white LED).

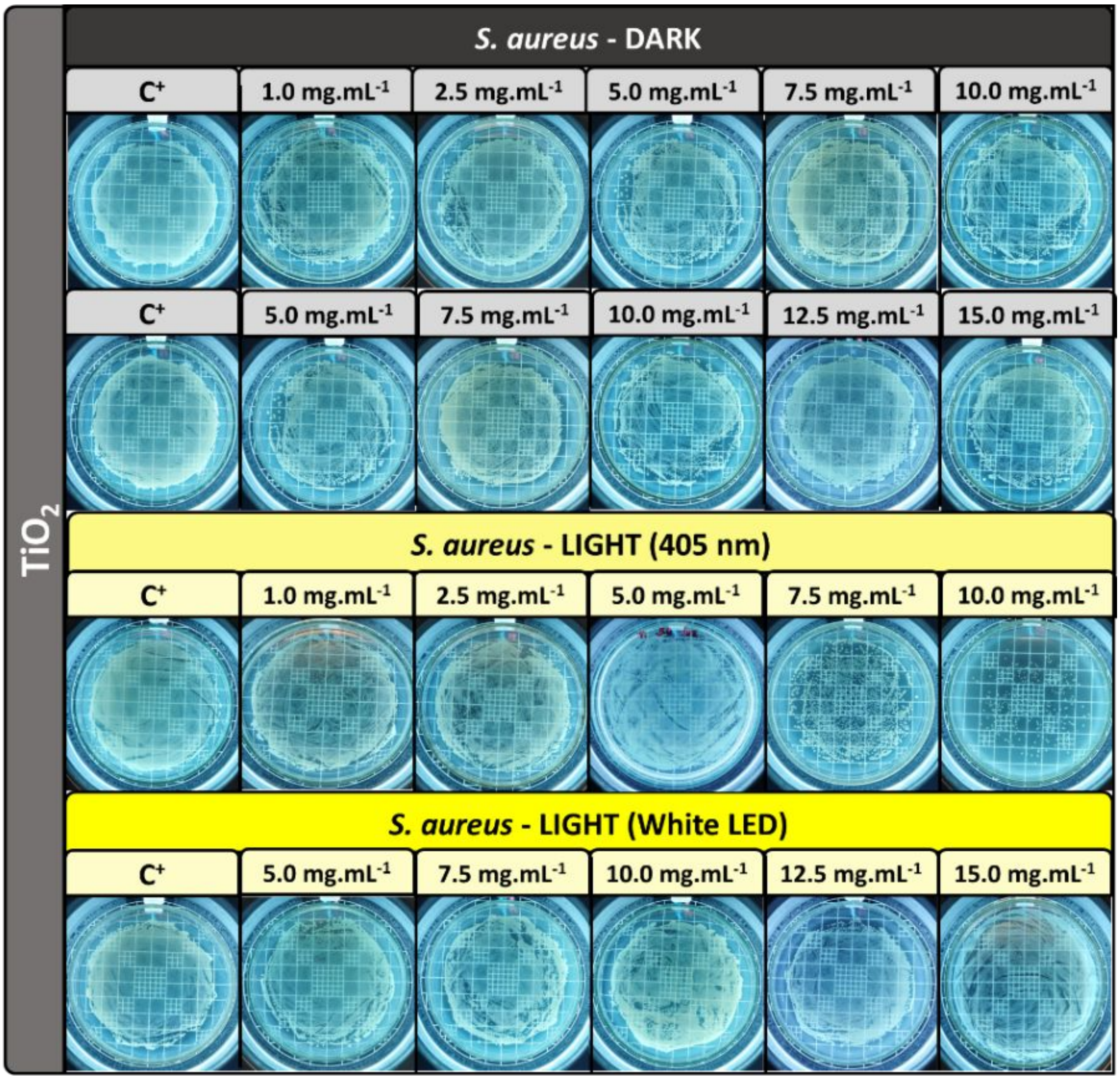

Fig. S6. MBC assay with S. *aureus* for pristine TiO_2 anatase NPs in dark and visible light conditions (405 nm and white LED).

Table S2. MIC and MBC values for Cu30-TiO_2 and Cu60-TiO_2 samples under dark or visible conditions.

Treatment	Bacteria	MIC ($mg.mL^{-1}$)	MBC ($mg.mL^{-1}$)	Dark/light
Cu30-TiO_2	*E. coli*	20.0	-	dark
Cu30-TiO_2	*E. coli*	10.0	12.5	405 nm LED
Cu30-TiO_2	*E. coli*	17.5	25.0	white LED
Cu30-TiO_2	*S. aureus*	12.5	-	dark
Cu30-TiO_2	*S. aureus*	5.0	7.5	405 nm LED
Cu30-TiO_2	*S. aureus*	10.0	15.0	white LED
Cu60-TiO_2	*E. coli*	17.5	25.0	dark
Cu60-TiO_2	*E. coli*	10.0	10.0	405 nm LED
Cu60-TiO_2	*E. coli*	17.5	22.5	white LED
Cu60-TiO_2	*S. aureus*	10.0	15.0	dark
Cu60-TiO_2	*S. aureus*	5.0	5.0	405 nm LED
Cu60-TiO_2	*S. aureus*	7.5	10.0	white LED

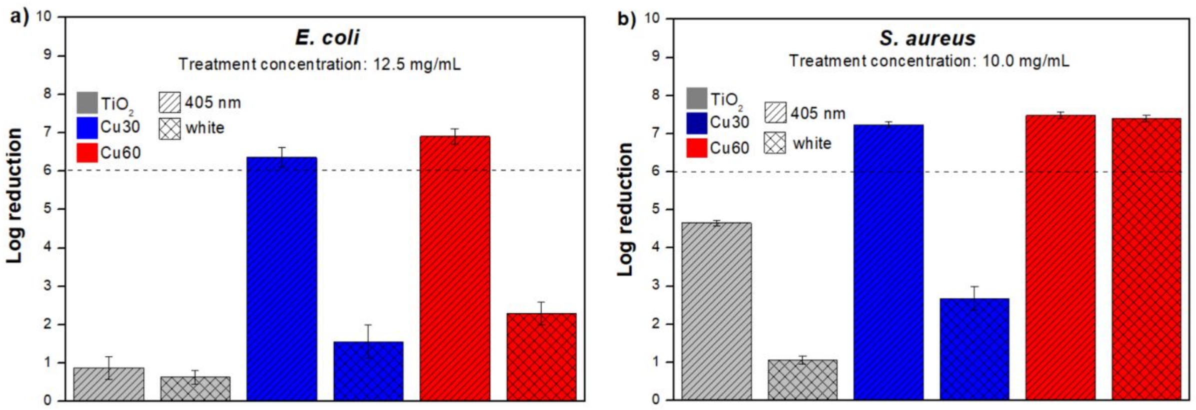

Figure S7. Log reduction graphs for TiO_2, Cu30-TiO_2 and Cu60-TiO_2 for *E.coli* (a) and *S. aureus* (b) under visible light conditions. The log reduction results were obtained in comparison with the control (without nanomaterial and under light illumination). The dashed lines are guides to the eye.
